# Sustainable Phosphorus Loadings from Effective and Cost-Effective Phosphorus Management Around the Baltic Sea

**DOI:** 10.1371/journal.pone.0005417

**Published:** 2009-05-04

**Authors:** Andreas C. Bryhn

**Affiliations:** Uppsala University, Department of Earth Sciences, Uppsala, Sweden; Cairo University, Egypt

## Abstract

Nutrient over-enrichment of the Baltic Sea, accompanied by intensified algal blooms and decreasing water clarity, has aroused widespread concern in the surrounding countries during the last four decades. This work has used a well-tested dynamic mass-balance model to investigate which decrease in total phosphorus loading would be required to meet the environmental goal to restore the trophic state in the Baltic Sea to pre-1960s levels. Furthermore, the extent to which various abatement options may decrease the phosphorus loading in a cost-effective manner has been studied. Upgrading urban sewage treatment in the catchment could, alone or in combination with banning phosphates in detergents, be sufficient to meet the set environmental goal, at an estimated annual basin-wide cost of 0.21–0.43 billion euro. Such a plan would potentially decrease the total phosphorus loading to the Baltic Sea with 6,650–10,200 tonnes per year.

## Introduction

Eutrophication as a result of anthropogenic over-enrichment of nutrients is a major environmental concern around the Baltic Sea, Northern Europe [1], [2]. The Baltic Sea shores are shared by nine countries (Denmark, Estonia, Finland, Germany, Latvia, Lithuania, Poland, Russia and Sweden) and long stretches of these shores serve as residential areas and popular summer holiday spots. Intensified cyanobacterial nuisance blooms and low Secchi depth (water clarity) are two obvious and widely acknowledged signs of eutrophication in the area. The annual basin-wide willingness-to-pay for restoring the ecological state of the Baltic Sea to conditions prior to the 1960s has been estimated at 3.6 billion euro (from [3], in 2008 prices).

**Figure 1:**
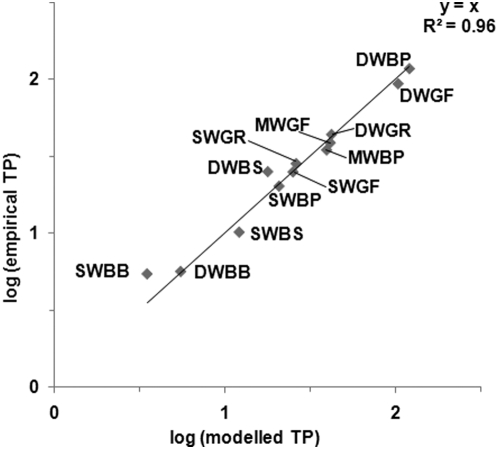
Log-transformed modelled and empirical annual mean TP concentrations (in µg/l). Data from Håkanson and Bryhn (2008). SW = surface waters, MW = middle waters, DW = deep waters. BP = Baltic Proper, BB = Bothnian Bay, BS = Bothnian Sea, GF = Gulf of Finland, GR = Gulf of Riga. Non-transformed annual and monthly values rendered r^2^ values of 0.95 and 0.84, respectively.

The total phosphorus (TP) loading to the Baltic Sea before the 1960s is poorly known. Regarding the difference between present loadings and those of the late 19^th^ or early 20^th^ century, estimates have decreased dramatically over time due to methodological improvements. An early estimate from Larsson et al. [4] indicated that the TP loading had increased by a factor of 8 since before the 20^th^ century. The development of dynamic nutrient models allowed Schernewski and Neumann [5] to conclude that the loading had increased over the past century by a factor of 4.5, while Savchuk et al. [2] calculated a three-fold increase. Håkanson and Bryhn [6] presented the first dynamic TP model with a unitary set of calibration constants which also delivered good predictions for all of the five major basins of the Baltic Sea (see Figure 1), and estimated that the TP loading had increased with about 50%.

Some researchers [7], [8] have hypothesised that both TP and nitrogen emissions affect TP concentrations as a part of a “vicious circle” in which nitrogen-limited algae die and sink towards deep sediments and when these algae are decomposed, dissolved oxygen is consumed. The resulting hypoxia in deep waters is assumed to intensify the diffusion of dissolved phosphorus from deep sediments to deep waters and eventually also to surface waters. However, the high levels of, and the great variations in, phosphorus concentrations in deep waters may have several explanatory factors, and the primary factor may be variations in oxygen-rich saltwater inflow from Kattegat, which connects the Baltic Sea to the North Sea and the Atlantic [9]. Furthermore, nitrogen modelling has not yet developed to the same stage as TP modelling, partly because many major nitrogen fluxes are highly variable and uncertain, which makes effects from nitrogen emissions more unpredictable than effects from phosphorus emissions [6]. The cyanobacterial community benefits from TP loadings but does not directly depend on anthropogenic nitrogen emissions since the dominating cyanobacterial species in the Baltic Sea can fix dissolved gaseous nitrogen of atmospheric origin [1].

Thus, while there is a widely accepted view that TP abatement is needed to increase the Secchi depth and decrease cyanobacterial blooms, nitrogen abatement is much more controversial since it may actually strengthen the competitiveness of cyanobacteria and increase the already massive nitrogen fixation flux to the ecosystem [1], [6], [10]. SANBALTS, a widely cited nitrogen and phosphorus model, which does not include a unitary set of model constants but requires basin-specific calibration, has recently been used to predict that the concentration of bioavailable (dissolved inorganic) nitrogen is similar or even lower today compared to one century ago, despite dramatic increases in nitrogen loading [2]. Phosphorus abatement alone has had demonstrated effects on algal bloom decreases in the Stockholm Archipelago of the Northern Baltic Proper [10] while no such system-wide effects have been possible to exclusively attribute to nitrogen abatement in the Baltic Sea or parts of it [1]. Therefore, the present work focuses on cost-effective phosphorus abatement.

Cost-effective environmental management is the art of reaching environmental targets at the minimal cost [11]. Such management may bring substantial benefits, and not only to those who care about monetary resources. Cost-effectiveness also means that the environmental “job” can be done as quickly as possible - i.e., that the time period during which considerable environmental harm is allowed is shortened to a minimum, since less expensive action is easier to take than expensive action. Cost-effectiveness should therefore be of great concern to most people who are interested in a clean environment and in well-functioning ecosystems.

Ideally, TP abatement measures should thus be 1) effective (decrease the loading with a large number of tonnes per year), 2) cost-effective (have a low marginal cost for each abated kilogram or tonne) and 3) politically feasible to ensure that measures are quickly adopted into legislation and policy. Previous work [2], [5], [6] has aimed at restoring conditions to those prevailing one century ago, but none of these studies have combined their findings with optimising the effectiveness and cost-effectiveness of detailed measures needed for restoration. Ambitious cost-effectiveness studies such as [11], [12], [13] and [14] have not specified which detailed measures would be required to reach a set environmental goal for the Baltic Sea. The aim of the present study is to determine which cost-effective abatement options may be effective enough to bring back the Secchi depth and cyanobacterial blooms to their pre-1960s level. The amount of TP which would need to be removed from the loading will also be assessed in order to produce data which are compatible with published estimates of the willingness-to-pay for environmental measures.

## Materials and Methods

For the time before 1960, data on TP loadings and TP concentrations are at best very scattered although detailed surveys are available for more recent decades [6], [9]. Regarding cyanobacteria, basin-wide information is for recent years mainly available as qualitative satellite images [15] although a historical review by [16] gives at hand that cyanobacteria were sometimes abundant but were not seen as an environmental problem before the late 1960s. Plenty of historical Secchi depth data are, however, freely available from Aarup's [17] database at www.ices.dk (accessed 2009-01-02). The trophic state of the Baltic Sea will in this work be assumed to have changed similarly with respect to both Secchi depth and cyanobacterial concentration. The investigated period includes the summer months (June to August) since this is the season when cyanobacterial blooms and other signs of eutrophication are usually the most disturbing and harmful [6], [15].

Of the variety of different TP models for the Baltic Sea, CoastMab, which will be used here, is the only published TP model which fulfils all of the following criteria [6]: 1) it takes into account all major TP fluxes to, from and within the Baltic Sea (inflow, outflow, sedimentation, diffusion, resuspension, erosion from land uplift and burial), 2) it has a unitary set of model constants and equations; i. e., these are the same for all basins, and 3) it gives good predictions of TP concentrations in all of the major basins of the Baltic Sea (Figure 1). Two comparable dynamic eutrophication models are ERGOM [5] and SANBALTS [2]. ERGOM does not fulfil criterion number 3 while SANBALTS does not fulfil criterion number 2; i. e., the latter model contains basin-specific algorithms and is tuned differently for different basins. This implies that the algorithms and constants of ERGOM and SANBALTS may not be able to properly capture general patterns of all major phosphorus fluxes which affect the Baltic Sea [6].

CoastMab predicts TP concentrations dynamically and, in addition, it predicts Secchi depths and cyanobacteria from empirical cross-systems regressions. Details are given in [6], including previous additional application sites for the model (Baltic Sea coastal areas and Ringkøbing Fjord, Denmark). Nitrogen concentrations, salinities and temperatures will be assumed to remain stationary at present levels during simulations in this work.

Figure 1 illustrates that there are some uncertainties in CoastMab's predictions and in empirical TP data (see [6] for a detailed discussion about these uncertainties). The cross-systems validated sub-models for the Secchi depth and cyanobacteria in CoastMab as well as the empirical Secchi data in this work will most likely add even more uncertainty to comparisons between Secchi depth predictions and observations. These combined uncertainties will be used to calculate the uncertainty in the required decreases in TP loading.

The marginal cost for TP abatement is the cost for removing one kg (or another mass unit) of TP from the loading to the Baltic Sea. Marginal costs of various TP abatement options will be taken from [11], [12], [13], [14] and [18]. All costs will be transformed into 2008 prices using official statistics of Swedish and euro-area consumer price indices. Swedish crowns (at 2008 prices) will be converted to euro at the rate 10∶1 and all prices will thus be expressed in euro at their 2008 value.

## Results and Discussion

### Secchi depths, cyanobacterial concentrations, and phosphorus loadings

**Figure 2:**
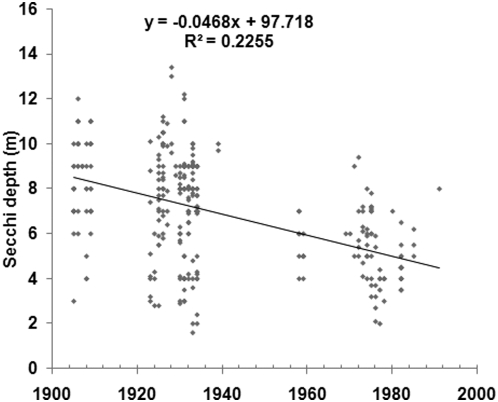
The Secchi depth in the Gulf of Finland, June–August, 1905–1991. Number of data: 315.

**Figure 3:**
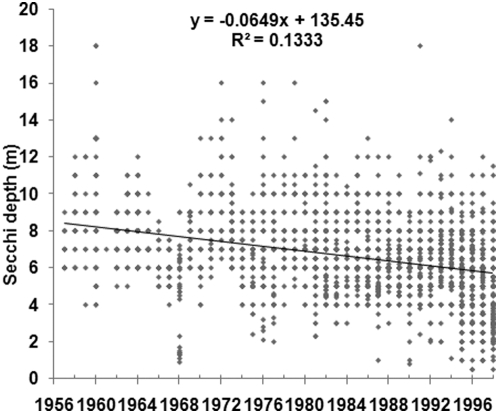
Secchi depth in the Baltic Proper, June–August, 1957–1998. Number of data: 3,452.

Historical Secchi depth measurements from before 1960 were available for two major basins of the Baltic Sea; the Gulf of Finland and the Baltic Proper. Figure 2 shows the Secchi depth during June to August in the Gulf of Finland since year 1900, and regression analysis, a t-test and a non-parametric test all showed that the Secchi depth has decreased significantly over time, although the variability of the data was substantial. From the early 20^th^ century, the Secchi depth decreased from a median value of 9.0 m to a median value of 5.0 m 1980–1991. There were few data from the years immediately preceding 1960 (the median Secchi depth 1958–1959 was 5.0 m) and although the mean Secchi depth during 1958–1959 was slightly higher than 1980–1991 (5.41 m compared to 4.92 m), the difference was not statistically significant at the 95% confidence level. Figure 3 and Table 1 shows the Secchi depth of the growing season in the Baltic Proper 1957–1998 and there were plenty of data from this whole period to confirm that the Secchi depth has decreased significantly over time, from 8.0 m 1957–1959 to 5.5 m 1995–1998. As a comparison, the median Secchi depth was 10.0 m (mean value 9.78 m; 93 measurements) 1903–1909.

**Table 1:** Secchi depth (in m) in the Baltic Proper 1957–1998.

Period	Mean	Median	Standard deviation	Number of data
1957–1959	8.12	8.00	1.72	57
1960–1964	8.14	8.00	1.86	325
1965–1969	6.56	7.00	1.66	187
1970–1974	8.02	8.00	2.01	195
1975–1979	7.11	7.00	2.21	265
1980–1984	7.04	7.00	1.87	463
1985–1989	6.56	6.00	1.60	620
1990–1994	6.46	6.25	1.70	650
1995–1998	5.42	5.50	1.93	690

Since the 1990s, the Secchi depth has decreased further in the Gulf of Finland, the Northern Baltic Proper and the Western Gotland Basin, while mean values have stabilised in the Eastern Gotland Basin and the Secchi depth has recovered to the 1970s levels or even further in the South–Western Baltic Sea (the Arkona Sea and the Bornholm Sea [19]). However, individual observation data from [19] were not available to the author, so for model simulations, data from the Baltic Proper were used because of good data availability and because of a documented significant decrease in Secchi depth in this basin since the late 1950s.

**Figure 4:**
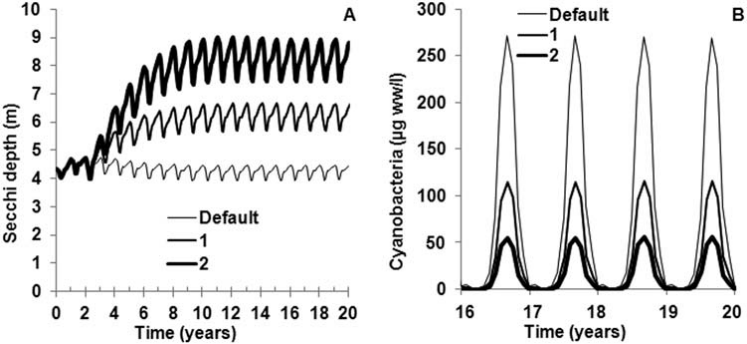
Modelled Secchi depths and cyanobacterial concentrations at late 1990s TP loadings (default), during a 6,650 tonne decrease of the annual TP loading after two years (scenario 1) and during a 10,200 tonne decrease of the annual TP loading after two years (scenario 2).

Figure 4 shows modelling results for three different scenarios in the Baltic Proper, with the default scenario describing TP loadings during a typical year in the late 1990s. Thus, results describe average conditions; annual variations in cyanobacterial bloom intensity may be particularly substantial [6]. Secchi depths (Figure 4A) were lower in this scenario than in Table 1 and Figure 3, which can be attributed to model uncertainties and uncertainties in empirical data. A 45% increase in Secchi depth (corresponding to the relative empirical difference in median values between 1995–1998 and 1957–1959) is displayed scenario 1 in Figure 4, and an annual TP loading decrease of 6,650 tonnes to the three most heavily loaded basins (the Gulf of Finland, the Gulf of Riga and the Baltic Proper) was required to drive this model scenario. To predict median summer Secchi depths of 8.0 m (as during 1957–1959; Table 1), a 10,200 tonne decrease of the annual TP loading was required and the results are displayed as scenario 2 in Figure 4. The summer mean concentration of cyanobacteria decreased sharply during the two remedial scenarios; with about 60% during scenario 1 and about 80% during scenario 2. It should, however, be stressed that decreased nitrogen concentrations or increased surface water temperatures could offset such decreases in cyanobacterial concentrations in the Baltic Proper [15].

### Effectiveness and cost-effectiveness of abatement measures

How many tonnes of TP can be removed from the loadings to the Baltic Sea by means of various abatement measures covered in the literature specified in Materials and Methods is displayed in Table 2. Upgrading sewage treatment may be inevitable to fulfil the requirements for scenario 2 (10,200 tonnes TP removed from annual inputs) and would be sufficient for achieving both scenarios 1 (6,650 tonnes TP removed per year) and 2. Agricultural measures and banning phosphates in detergents could (according to Table 2) be two other options with substantial effects on the TP loading which, in combination, could be sufficient for scenario 1 but insufficient for scenario 2. Some other measures, such as cultivating and harvesting mussels in Swedish waters and constructing Swedish wetlands, would make small differences to the loading.

**Table 2:** Possible measures for decreasing the phosphorus input to the Baltic Sea.

Measure	Area	Tonnes/year
Sewage treatment	Poland	5,332
Sewage treatment	Russia	3,844
Sewage treatment	Belarus	1,984
Sewage treatment	Baltic States	992
Sewage treatment	Czech Rep.	372
Phosphate-free detergents*	All	3,100
Agriculture	All	5,600
Rural sewage treatment	Sweden	175
Mussel cultivation	Sweden	35
Dams	Sweden	10
Protective zones	Sweden	7.5
Wetlands	Sweden	4.3
Soil drainage	Sweden	2.6

* In combination with sewage treatment.

Swedish examples are from [18] and the other examples emanate from [27].

Table 3 shows some marginal costs for TP abatement in connection with measures in agriculture, sewage treatment and wetland construction. Costs were taken from [11] and survey data are thus to some extent outdated since some of the measures have already been implemented, but the important message from this table regards which marginal costs these three different abatement strategies commonly have in relation to each other. Marginal costs in sewage treatment plants (STPs) were consistently lower in all regions listed in Table 3 than any of the costs for measures in agriculture or wetland construction. Agricultural measures were a factor of 5–50 more expensive and costs for removing phosphorus in wetlands were a factor of 25 more costly than sewage treatment. Removing phosphorus in Swedish wetlands would generally be the least cost-effective option according to Table 3.

**Table 3:** Marginal abatement costs (euro per kg; 2008 prices) for phosphorus to the Baltic Sea.

Region	Agriculture	STPs	Wetlands
Sweden	18–772	4.8–6.1	2,133
Finland	26–711	4.8–6.1	204
Denmark	17–305	4.8–8.0	141
Germany	22–347	4.8–8.0	105
Poland	13–238	2.3–12	71
Estonia	33–658	2.3–12	712
Latvia	27–662	2.3–12	144
Lithuania	22–783	2.3–12	113
St Petersburg	27–505	2.3–12	96
Kaliningrad	40–502	2.3–12	64

STPs = sewage treatment plants. From [11].

The most relevant marginal cost data for this study from [13] are listed in Table 4. Wetland construction generally had low cost-effectiveness (in correspondence with Table 3), with the exception of Lithuanian wetlands (contrary to the information in Table 3), although there are no data in [13] on how many tonnes of TP could be abated by creating new Lithuanian wetlands. In addition to high construction costs, the general long-term effectiveness of wetlands as phosphorus sinks is quite uncertain [20]. Sewage treatment would be more cost-effective than wetlands, and treatment in urban settlements in the eastern part of the catchment when no additional pipes need to be constructed would be particularly cost-effective (20 euro per kg according to Table 4). Some examples regarding urban STPs near the eastern shores are specified in [12] where chemical phosphorus treatment in functioning STPs could potentially remove 520 tonnes P per year at a marginal cost of 8 euro/kg; constructing the South-Western STP in St. Petersburg (Russia) would remove 200 tonnes/year at 35 euro/kg while constructing the 12 km long Northern sewage collector in the same area would remove 220 tonnes/year at 43–88 euro/kg phosphorus. These figures were compared with much less cost-effective measures in Finnish agriculture at an average marginal cost of 220 euro/kg phosphorus [12].

**Table 4:** Marginal abatement costs (euro per kg) for phosphorus to the Baltic Sea. From [13].

Measure	Area	Marginal abatement cost
Wetlands	Germany	103
Wetlands	Denmark	170
Wetlands	Estonia	153
Wetlands	Finland	92
Wetlands	Lithuania	35
Wetlands	Latvia	142
Wetlands	Poland	73
Wetlands	Russia	643
Wetlands	Sweden	163
Urban sewage treatment, no pipes	Eastern basin	20
Urban sewage treatment, including pipes	Eastern basin	42
Rural sewage treatment, no pipes	Eastern basin	43
Rural sewage treatment, including pipes	Eastern basin	92
Urban sewage treatment, no pipes	Western basin	30
Urban sewage treatment, including pipes	Western basin	63
Rural sewage treatment, no pipes	Western basin	65
Rural sewage treatment, including pipes	Western basin	138
Phosphate-free detergents	Denmark	44
Phosphate-free detergents	Estonia	19
Phosphate-free detergents	Finland	39
Phosphate-free detergents	Lithuania	14
Phosphate-free detergents	Latvia	19
Phosphate-free detergents	Poland	18
Phosphate-free detergents	Russia	13
Phosphate-free detergents	Sweden	53
Less milk cows	Germany	14
Less milk cows	Denmark	20
Less milk cows	Estonia	13
Less milk cows	Finland	19
Less milk cows	Lithuania	4.1
Less milk cows	Latvia	12
Less milk cows	Poland	10
Less milk cows	Russia	13
Less milk cows	Sweden	12
Less pigs	Germany	15
Less pigs	Denmark	18
Less pigs	Estonia	22
Less pigs	Finland	18
Less pigs	Lithuania	6.2
Less pigs	Latvia	19
Less pigs	Poland	11
Less pigs	Russia	12
Less pigs	Sweden	14

Banning phosphates in detergents seems quite cost-effective according to Table 4, although attention must be paid to the regional differences. In Sweden, where sewage treatment has been implemented with relatively ambitious standards, marginal costs are actually higher than most sewage treatment alternatives in Table 4 and this would probably be the case for more countries if they would first upgrade their sewage treatment to Swedish standards. Vice versa, if phosphates in detergents have already been banned, then constructing STPs would be less cost-effective than if phosphates would be allowed in detergents. This means that if the goal would be to remove 3,100 tonnes of phosphorus per year, then a phosphate ban in detergents would be a cost-effective option according to Table 2 and Table 4. If more than 12,400 tonnes of P should be removed from yearly loadings, sewage treatment together with a phosphate ban could also be cost-effective. However, if the abatement goal lies well above 3,100 but below 12,400 tonnes/year, as would be the case for scenario 2 in this work, upgrading sewage treatment to Swedish standards without banning phosphates in detergents could actually be both sufficient and the most cost-effective option according to Table 2 and Table 4. It should also be noted that alternatives to phosphates in detergents may have their own adverse environmental effects. One of the most viable alternatives, Zeolite A, produces greater volumes of sludge which cannot be recycled in the same manner as phosphorus in sewage sludge can be used as a fertiliser in agriculture. Thus, with effective sewage treatment in place, phosphates may actually be the most environmentally friendly option in a life-cycle perspective [21] which would imply much lower cost-effectiveness for a phosphate ban than what can be extracted from Table 4.

The effects on the TP loading and the marginal cost with respect to most available agricultural measures are not specified in [13] due to a reported lack of data. One exception is the possibility to reduce the livestock (milk cows and/or pigs), which would have comparatively low marginal costs in most countries (Table 4). This measure includes replacing the manure from the animals with artificial fertiliser on the fields. How many tonnes of TP would then actually be prevented from reaching the Baltic Sea as a result of this measure is unfortunately not specified (directly or via proper references) in [14]. Relationships between changes in land use and TP loadings to the sea are generally inadequately researched and sometimes weak, obscure or even contradictory [22]. The TP content in soils may change very slowly even after drastic changes in land use [23]. In other words, it is unclear whether there would be any effects at all from this measure, or whether any substantial effects would require such radical measures (e.g., removing 80% of the livestock which was the upper limit for model simulations for nitrogen abatement in [24]) that inaction would be a much more palatable option for policymakers who wish to keep and extend their contracts. In addition, decreasing meat production without enforcing any changes in eating habits among the population could have the effect that local meat production would be substituted with imported meats, so that the eutrophication problem would instead be exported to other waters beyond the catchment of the Baltic Sea. Thus, although marginal costs may be low, the feasibility of eliminating meat production to decrease the TP loading appears to be low nonetheless when reliable alternatives such as improving sewage treatment or phosphate bans are readily at hand. Livestock reductions and most other agricultural measures investigated by [14], except for modest (no amount specified) reductions in fertiliser application, were less or much less cost-effective than urban sewage treatment and phosphate free detergents.

Most of the Swedish measures in Table 2 had low or very low cost-effectiveness compared to sewage treatment in Table 4. For instance, rural sewage treatment in Sweden had a marginal cost of 770–3,000 euro/kg [18]. The only exception among Swedish measures in Table 2 with potentially high cost-effectiveness is subsidised mussel cultivation and harvesting whose marginal costs would be 35 euro/kg [18], although since the potential impact is very small (35 tonnes/year; Table 2) it is disputable whether this option is worthwhile to develop beyond its present stage.

Instead, the most cost-effective plan to meet scenario 2 in Figure 4 would primarily include urban sewage treatment and Table 4 gives at hand that the yearly cost of such a plan would probably not exceed 0.43 billion euro per year (10,200 tonnes/year · 42 euro/kg · 1000 kg/tonne). Fulfilling scenario 1 in a cost-effective manner could include banning phosphates in detergents (3,100 tonnes/year; Table 2), at the median marginal cost (19 euro/kg) of the countries listed in Table 4, in addition to upgrading sewage treatment of TP with 3,550 tonnes/year at a total cost of 0.21 billion euro per year (3,100 tonnes/year · 19,000 euro/tonne+3,550 tonnes/year · 42,000 euro/tonne). Scenarios 1 and 2 are then assumed to involve the same marginal cost for TP abatement in sewage treatment; more expensive treatment measures would be required for scenario 2 but marginal costs for sewage treatment would increase in scenario 1 since phosphates in detergents would also have been banned and would not be removed in STPs. These cost estimates are somewhat lower than those of [14] which would correspond to about 0.3 and 0.5 billion euro per year for scenarios 1 and 2, respectively (17% and 26% reductions of 38,900 tonnes per year to the Baltic Sea, the Belt Sea and Kattegat). Regardless of which one of these cost estimates is the most accurate one, all of them are much lower than the estimated yearly cost of the recently adopted Baltic Sea Action Plan, at 3.1 billion euro per year [13]. This plan would require some nitrogen and phosphorus abatement options with low cost-effectiveness. Since the willingness-to-pay stated in the Introduction (3.6 billion euro) concerns other environmental problems in the Baltic Sea in addition to eutrophication (e.g., overfishing, oil spills, organic toxins and other contaminants, and invasions of alien species), it is questionable whether the Baltic Sea Action Plan will have public support once the full costs and the other necessary societal efforts will clarify. However, the abatement plans motivated in this work could leave plenty of consumer surplus to be funnelled into action against other environmental threats than eutrophication.

An additional advantage of the presented plan is that it addresses point sources of TP rather than diffuse sources. Inputs from point sources have a rather low variability over the year but inputs from diffuse sources, which are transported by rivers, tend to peak in spring [25] while cyanobacteria primarily bloom during the summer [16]. Riverine TP loading to the Baltic Sea is about 60% greater from March to May than from June to August [26]. However, the TP flux to surface waters from shallow sediments and deeper waters may be a factor of 8 larger than inputs from land [6]. These internal fluxes also reflect loadings from previous seasons and years, so the advantage of addressing point sources is probably greater in terms of cost-effectiveness than in the timing of inputs.

### Conclusion

This work has investigated TP loading requirements and abatement costs for restoring the trophic state (Secchi depth and cyanobacterial conce ntration) of the Baltic Sea to pre-1960s levels. Simulations with the CoastMab model showed that 6,650–10,200 tonnes of TP would have to be removed from the annual loadings to meet this goal; i.e., to increase the Secchi depth in the Baltic Proper with 45% (from 5.5 to 8.0 m as median values) during the summer months.

TP abatement measures in agriculture and wetland construction were found to have low cost-effectiveness compared to other options. Agricultural measures which imply decreased production due to changes in land use should be avoided because they have uncertain effects on the TP loading, they could be difficult to raise public and political support for, and they could also export the eutrophication problem to other regions of the world while increasing food transports.

There were, however, two promising investigated action alternatives which may lead to substantial, cost-effective decreases in TP loading, and these are upgrading urban sewage treatment in the eastern part of the catchment, and banning phosphates in detergents. The total abatement cost for applying one or two of these measures to the extent that the trophic state would be restored to conditions prevailing before 1960 was estimated at 0.21–0.43 billion euro per year. This cost estimate for removing 6,650–10,200 tonnes of TP per year was 17–30% lower than the estimate in [14]. The total cost is particularly sensitive to the extent to which additional pipes need to be constructed and connected to sewage treatment plants, to how much phosphorus can actually be removed from urban wastewater with Swedish standards on sewage treatment, and to the life-cycle cost effectiveness of banning phosphates in detergents.
